# Wei Chang An pill regulates gastrointestinal motility in a bidirectional manner

**DOI:** 10.1080/13880209.2021.1991383

**Published:** 2021-10-28

**Authors:** Sitong Jia, Lijuan Chai, Jing Zhang, Min Zhang, Lin Li, Yaxin Qi, Yafen Pang, Xi Chen, Nana Fan, Lin Wang, Yujing Wang, Jixiang Song, Yingjie Sun, Yi Wang, Lin Miao, Han Zhang

**Affiliations:** a Institute of Traditional Chinese Medicine, Tianjin University of Traditional Chinese Medicine, Tianjin, China; b State Key Laboratory of Component-based Chinese Medicine, Tianjin University of Traditional Chinese Medicine, Tianjin, China; c Key Laboratory of Pharmacology of Traditional Chinese Medical Formulae, Ministry of Education, Tianjin University of Traditional Chinese Medicine, Tianjin, China; d Laboratory of Pharmacology of TCM Formulae Co-Constructed by the Province-Ministry, Tianjin University of TCM, Tianjin, China; e Tianjin Zhongxin Pharmaceutical Group Co., Ltd. Le Ren Tang Pharmaceutical Factory, Tianjin, China

**Keywords:** Diarrhoea, constipation, Rho-associated coiled-coil forming protein kinase-1 (ROCK-1), myosin light chain kinase (MLCK), irritable bowel syndrome (IBS)

## Abstract

**Context:**

Wei Chang An (WCA) is a commercial prescription developed for the coordination of gastrointestinal movement.

**Objective:**

To investigate the role of WCA in the regulation of diarrhoea and constipation in rats.

**Material and methods:**

The diarrhoea and constipation models were prepared by gavage of *Folium senna* and diphenoxylate hydrochloride. Rats were randomized equally (*n* = 6) into the normal group given saline daily, the positive group given Pinaverium Bromide (13.5 mg/kg) or Sennoside A (0.1 mg/kg) and three WCA-treated groups (22, 44, and 88 mg/kg) by gavage daily for 7 consecutive days. The effects of WCA were assessed by a series of faecal symptoms and histopathology. Gastrointestinal parameters were determined by ELISA. The effect of WCA on gastrointestinal tissues was evaluated by strip assay. Expression of ROCK-1 and MLCK was measured by RT-PCR and Western blotting.

**Results:**

Data from Bristol stool form scale, diarrhoea index, visceral sensitivity, defaecation time, and intestinal propulsive rate showed that WCA protected rats against diarrhoea and constipation (*p* < 0.01). The up-regulation of Substance P and 5-hydroxytryptamine in diarrhoea rats and down-regulation of Substance P and vasoactive intestinal polypeptide in constipation rats were inhibited by WCA (*p* < 0.05). WCA stimulated the gastrointestinal strip contractions but inhibited ACh-induced contractions (*p* < 0.01). The decreased ROCK-1 and MLCK expression in diarrhoea rats and increased in constipation rats were suppressed by WCA (*p* < 0.01).

**Conclusions:**

WCA has both antidiarrhea and anti-constipation effects, suggesting its bidirectional role in gastrointestinal modulation, and providing evidence of WCA for irritable bowel syndrome treatment.

## Introduction

Irritable bowel syndrome (IBS) is a common but complex gastrointestinal disorder characterized by chronic or recurrent abdominal pain, bloating, and cramping gas. It has been reported that more than 5% of adults worldwide suffer from IBS, which seriously influences their quality of life (Ballou and Keefer 2016; Sperber et al. 2017; Whitehead et al. 2017). According to the faeces patterns, IBS is classified into three subgroups including diarrhoea predominant (IBS-D), constipation-predominant (IBS-C) and mixed bowel habit (IBS-M), all of which involve dysfunction of gastrointestinal smooth muscle contraction that plays an important role, but in a distinct or even opposite way (DuPont et al. 2014; Camilleri 2018). Moreover, patients with IBS may experience variation between periods of constipation and diarrhoea (Fashner and Gitu 2013). It is a complicated problem to identify an effective medication as the general treatment for IBS patients due to contrary symptoms. Currently, it has been accepted that the predominant or most troublesome symptoms are considered the main target for drug application (Ohman and Simren 2007). It may be difficult to make an accurate diagnosis in some cases and adjustment of the medicine type is required whenever necessary. It would be more convenient for IBS treatment if a drug could ameliorate both the over-contraction and insufficient contraction of gastrointestinal smooth muscle, which may be considered as the effective management approach that helps towards various symptom relief.

Wei Chang An (WCA) pill is a commercial drug approved by China Food and Drug Administration (CFDA) and has been widely used for gastrointestinal disorder treatment for decades. As the instruction described, WCA prescripts ten herbs including *Aucklandia lappa* Decne. (Compositae), *Aquilaria sinensis* (Lour.) Gilg (Thymelaeaceae), *Citrus aurantium* L. (Rutaceae) (also called Fructus Aurantii), *Magnolia offificinalis* Rehd. et Wils. (Magnoliaceae), *Santalum album* L. (Santalaceae), *Rheum offificinale* Baill. (Polygonaceae), *Croton tiglium* L. (Euphorbiaceae), *Moschus moschiferus* Linnaeus, *Ligusticum chuanxiong* Hort. (Umbelliferae) and *Ziziphus jujuba* Mill. (Rhamnaceae) (Liu et al. 2013). Previous studies have analyzed the chemical composition of WCA by high-performance liquid chromatography-tandem mass spectrometry (HPLC-MS) and high-performance liquid chromatography with diode array detector and electrospray ionization-tandem mass spectrometry (HPLC-DAD-ESI-MS/MS); 68 known chemical compounds were detected and 41 unknown compounds were identified (Liu et al. 2013; Zhang et al. 2013). A few reports have shown that WCA has definite effects in various gastrointestinal diseases. Clinical observation of 60 cases of functional diarrhoea showed that WCA treatment improved the symptoms of diarrhoea and the total effective rate up to 98.3% (Liu et al. 2016). Pharmacological research showed that administration of WCA significantly accelerated gastrointestinal transit in normal mice and reduced stimulated neostigmine-induced gastrointestinal transit (Hu et al. 2009). WCA also attenuated spontaneous contractions induced by ACh or neostigmine in rabbit jejunum and inhibited gastric emptying (Hu et al. 2009; Wang et al. 2012). It has also been noticed that WCA exhibited a bidirectional effect on gastrointestinal transit and the spasmolytic activity of rat-isolated jejunum (Qu et al. 2014). However, it remains unclear that whether WCA ameliorates both diarrhoea and constipation and how WCA balances the contractile behaviours along the gastrointestinal tract.

In our study, to detect whether WCA has a dual effect on intestinal motility responses, the effects of WCA on *Folium senna* [*Cassia angustifolia* Vahl. (Leguminosae)] induced-diarrhoea rats and diphenoxylate hydrochloride (CDT) induced-constipation rats were assessed separately, and the strip contraction and the contractile-related gene expressions were also analyzed, which give evidence of WCA on the improvement of the abnormal gastrointestinal motility in a proper manner.

## Materials and methods

### Chemicals and reagents

WCA was supplied by Le Ren Tang Zhongxin Pharmaceutical Group Co., Ltd. (Tianjin, China). *Folium senna* was bought from Liankang Pharmaceutical Co., Ltd. (Hebei, China). Diphenoxylate hydrochloride (CDT) was produced by Dingchang Pharmaceutical Co., Ltd. (Henan, China). Pinaverium Bromide Tablets (PBT) were obtained from Abbott Healthcare SAS Co., Ltd. (Shanghai, China). Sennosides A was obtained from Meilun Biotechnology Co., Ltd. (Dalian, China). Enzyme-linked immunosorbent assay (ELISA) kit of NO was obtained from Nanjing Jiancheng Bioengineering Institute (Nanjing, China). ELISA kits of Substance P (SP), motilin (MTL), 5-hydroxytryptamine (5-HT) and vasoactive intestinal polypeptide (VIP) were bought from Huamei Bioengineering Co., Ltd. (Wuhan, China).

### Preparation of extract

Commercial WCA pills (10 g) were first powdered and filtered through a 100-mesh screen. Then 1.136 L H_2_O was added to prepare the WCA extract at the concentration of 8.8 mg/mL at room temperature.

### Animals

Wistar male rats (seven-week-old, 250 ± 10 g) were bought from Charles River Laboratory Animal Technology Co., Ltd. (Beijing, China) and housed in a dedicated animal room and maintained at constant temperature (22 ± 5 °C) and humidity (55% ± 5%), allowing free access to food and water. The procedures were approved by Animal Research Committee (TCM-LAEC20170018) in accordance with the Guide for the Care and Use of Laboratory Animals.

### Diarrhoea model

Rats were divided randomly into six groups including normal control (NC), Diarrhoea group, three WCA groups (WCA-L, WCA-M, WCA-H) and PBT (*n* = 6). Except for the NC group, rats from the other five groups were fasted for 24 h and then administered with 20% *Folium senna* extract by gavage. Subsequently, rats were placed in breathable plastic bottles for 2 h. After restriction, rats in different groups were given equal volumes of water, PBT (13.5 mg/kg) or WCA (22, 44, 88 mg/kg) by gastric lavage.

### Bristol stool form scale

The faeces were collected in the filter paper which was previously inserted to the bottom of the cage. Faeces properties were scored as Bristol stool form scales according to cohesion and surface cracking described in Table 1 concerning the previous report (Koh et al. 2010).

**Table 1.** Grading scores of faecal properties.

Classification	Faecal property	Score
Level 1	Separate hard blocks (such as nuts)	1
Level 2	Sausage shaped, but with blocks	2
Level 3	Like sausage, but with cracks on the surface	3
Level 4	Like sausage, smooth and soft	4
Level 5	Soft spots, with transparent cutting edges	5
Level 6	Paste, with uneven edges	6
Level 7	No solid, liquid-like water	7

### Diarrhoea index

After diarrhoea modelling, each rat was placed in a cage with absorbent paper that was replaced hourly to record the defecations. Diarrhoea index was calculated as the loose faeces rate multiplied by the mean loose faces grade (Hu and Tang 2012). The loose faeces rate was calculated as the ratio of loose faeces number to the total faeces numbers produced by each animal. Each granule or each pile of faeces (when it was not possible to determine the number of granules) was regarded as one. Loose faeces were graded by the diameter of the stain formed by the faeces on the filter papers, in four degrees: <1 cm (grade 1), 1–2 cm (grade 2), 2–3 cm (grade 3), and >3 cm (grade 4). For faeces of elliptical or irregular shape, the longest diameter or an approximate round diameter were recorded. Mean loose faeces grade was calculated as the sum of loose faeces grade divided by the number of loose faeces.

### Visceral sensitivity

After seven-day administrations, visceral sensitivity was evaluated by rectal distension detection. Rats were placed in plastic channels (6 cm in diameter and 25 cm in length) so that they were unable to move, escape or turn around to prevent airbag damage. Then, shida double chamber catheter (Changde, Hunan, China) was inserted into the rat rectum from the anorectum, and kept inside for 6 cm. After fixation of catheter tail, water (1.2 mL) was injected into the catheter to expand the airbag gradually, which lasted 15 min once, 3 times in total at an interval of 5 min. During the period, the behaviour state, abdominal contraction amplitude and frequency were assessed. The scoring criteria of visceral sensitivity were described in detail in Table 2.

**Table 2.** Visceral sensitivity score.

Volume (ml)	Score
<0.6	5
0.79 − 0.6	4
0.99 − 0.8	3
1.19 − 1.0	2
≥1.2	1

### Constipation model

Rats were divided randomly into six groups including NC group (NC), Constipation groups, and three WCA groups (WCA-L, WCA-M, WCA-H) and sennosides A group (*n* = 6). Except for the NC group, rats from the other five groups were fasted for 24 h and were orally administered with CDT suspension (10 mg/kg) (Cong et al. 2019) once a day for 14 d to induce constipation. Then rats were given water or different doses of WCA (22, 44, 88 mg/kg) or sennosides A (0.1 mg/kg) for 7 days.

### Defaecation time

Six rats in each group were used to examine intestinal peristaltic function. All groups were fasted for 24 h with free access to water and received an oral administration of 0.2 mL of gastric ink (Jintangwenbao, Tianjin, China) every other day. Then the rats were placed in separate cages with free access to water and food. Six rats from each group were used to record the time until the defaecation of the first black faeces.

### Determination of total number and weight

The excreted faecal pellets of individual rats were collected every day throughout the duration of the experiment for 21 days. The total number and weight of the pellets were determined.

### Intestinal propulsive rate

After seven-day WCA treatment, rats were administrated with gastric ink and 25 min later the rats were sacrificed by decapitation under mild anaesthesia. The intestinal part from the pylorus to the ileocecal was cut off and its length was measured as the distance of the activated ink migration. The intestinal propulsive rate was calculated as follows: Intestinal propulsive rate = Distance of the activated ink migration/Whole length of intestine.

### Determination of hormone levels in serum

After anaesthetising, rat blood from the abdominal aorta was taken and centrifuged at 3000 rpm for 10 min at 4 °C, and the supernatant was collected as serum. Concentrations of SP, MTL, 5-HT and VIP were measured using ELISA kits (Huamei Bioengineering Co., Ltd., Wuhan, China). According to the manufacturer’s protocol, samples were pipetted into the microplates pre-coated with antibodies specific for SP, MTL, 5-HT and VIP respectively. After removing unbound substances, biotin-conjugated antibodies were added followed by washing and avidin conjugated Horseradish Peroxidase (HRP) incubation. After colour development, the intensities were measured at 450 nm. The level of NO in serum was measured by measurement of the total nitrate/nitrite level (Nanjing Jiancheng Bioengineering Institute, Nanjing, China). Nitrate reductase and enzyme cofactor were first added and incubated at room temperature for 1 h to convert nitrate to nitrite. Then, 5 µL enhancer was added. After 10 min incubation. Then 50 µL Griess reagents of R1 and R2 were added and OD value at 540 nm was then detected. The concentrations were calculated by a calibration curve prepared by standard concentrations as *X*-axis, and OD values as *Y*-axis.

### Microscopy imaging

After being isolated from rats, specimens of colon tissues were fixed in 10% formalin and embedded in paraffin, and 5 μm thick sections were cut and stained with hematoxylin-eosin (HE) as previously described (Miao et al. 2019). The histopathological images were visualized by microscope (Nikon, Tokyo, Japan). The five longest villi in each tissue section and a total of 90 longest villi were analyzed in each group. Villus height and crypts depth were measured using ImageJ software. The number of goblet cells per 100 enterocytes was calculated.

### Smooth muscle contraction assay

Isolated gastric antrum, jejunum or ileum muscle strips (6-10 mm × 2 mm) were mounted in separate 10 mL glass, water-jacketed organ baths, containing Krebs buffer (NaCl 118 mM, KCl 4.75 mM, KH_2_PO_4_ 1.2 mM, NaHCO_3_ 25 mM, glucose 11 mM, MgSO_4_ 1.2 mM and CaCl_2_ 1.8 mM) bubbled with 5% CO_2_ in O_2_, and maintained at 37 °C. One side of the muscle strips was ligated to the L-shaped tube with Krebs buffer and the other end was connected to the muscle tension sensor, which was used to tense the force produced by the tissue. The tissue was suspended under isotonic tension of 2 g and allowed to equilibrate for at least 10 min with Krebs buffer. The contraction of muscle strips was induced by the addition of ACh (final conc. 10 μM), and the effect of WCA has then evaluated upon WCA (final conc. 4 mg/mL) treatment. A Power Lab data acquisition system 8/30 (AD Instruments, Sydney, Australia) was used to process information and record the tension.

### RNA extraction and RT-PCR

Total RNA was isolated from rat antrum and duodenum using TRIzol reagent (CWBIO, Beijing, China) following the manufacturer’s protocol. The estimated RNA concentration was obtained by absorbance the value of A260 (ng/mL). The reverse transcription was then performed at 37 °C for 2 h, as described previously (Miao et al. 2019). The 20 μL reaction solution included 1–2 g of RNA, 0.5 mM of deoxy-NTPs, 5 M of random hexamers, 10 mM of dithiothreitol, 200 U Moloney murine leukaemia virus reverse transcriptase and 20 U ribonuclease inhibitor (all from Promega, Madison, WI). Real-time PCR was performed to determine the levels of mRNA for ROCK-1 by Go Tap-qPCR-Master-Mix (Promega, Beijing, China) according to the instructions. The relative gene expression was determined using the comparative CT method and normalized to a housekeeping gene GAPDH. The primers used are listed in Table 3 and were synthesized by Sangon (Shanghai, China).

**Table 3.** Primer sequences.

Genes	Primer sequences
ROCK-1	
Forward	5′-AGGCCTGTGCCAAACCTTT-3′
Reverse	5′-TGGTCCCTGTGGGACTTAACA-3′
GAPDH	
Forward	5′-ATGATTCTACCCACGGCAAG-3′
Reverse	5′-CTGGAAGATGGTGATGGGTT-3′

### Western blotting

Tissue proteins isolated from rat antrum and duodenum were extracted with RIPA buffer and quantified using the Bradford method. 20 μg of protein was loaded to each well of 10% SDS-PAGE, and then transferred to PVDF membrane, following by blocking with 5% milk, and then incubated with anti-ROCK-1 (Cell Signalling, MA, USA**)**, anti-MLCK (Abcam, Shanghai, China), anti-GAPDH (Proteintech, Wuhan, China) antibody at 4 °C overnight. After washing with TBST, the membrane was incubated with the secondary antibodies (Zhongshan Jinqiao, Beijing, China) for 1 h, and then was developed by enhanced chemiluminescence and visualized with Hyperfilm - ECL (General Electric, Piscataway NJ, USA). The band grey densities were quantified by Image J densitometry software.

### Statistical analysis

Data were expressed as means ± standard error. Statistical significance was analyzed with Student’s *t*-test and one-way analysis of variance (ANOVA) followed by Dunnett’s test. Tests were performed using SPSS 16.0 System. *p*-value < 0.05 was considered to be statistically significant.

## Results

### WCA inhibited *Folium senna*-induced diarrhoea in rats

**Figure 1.**
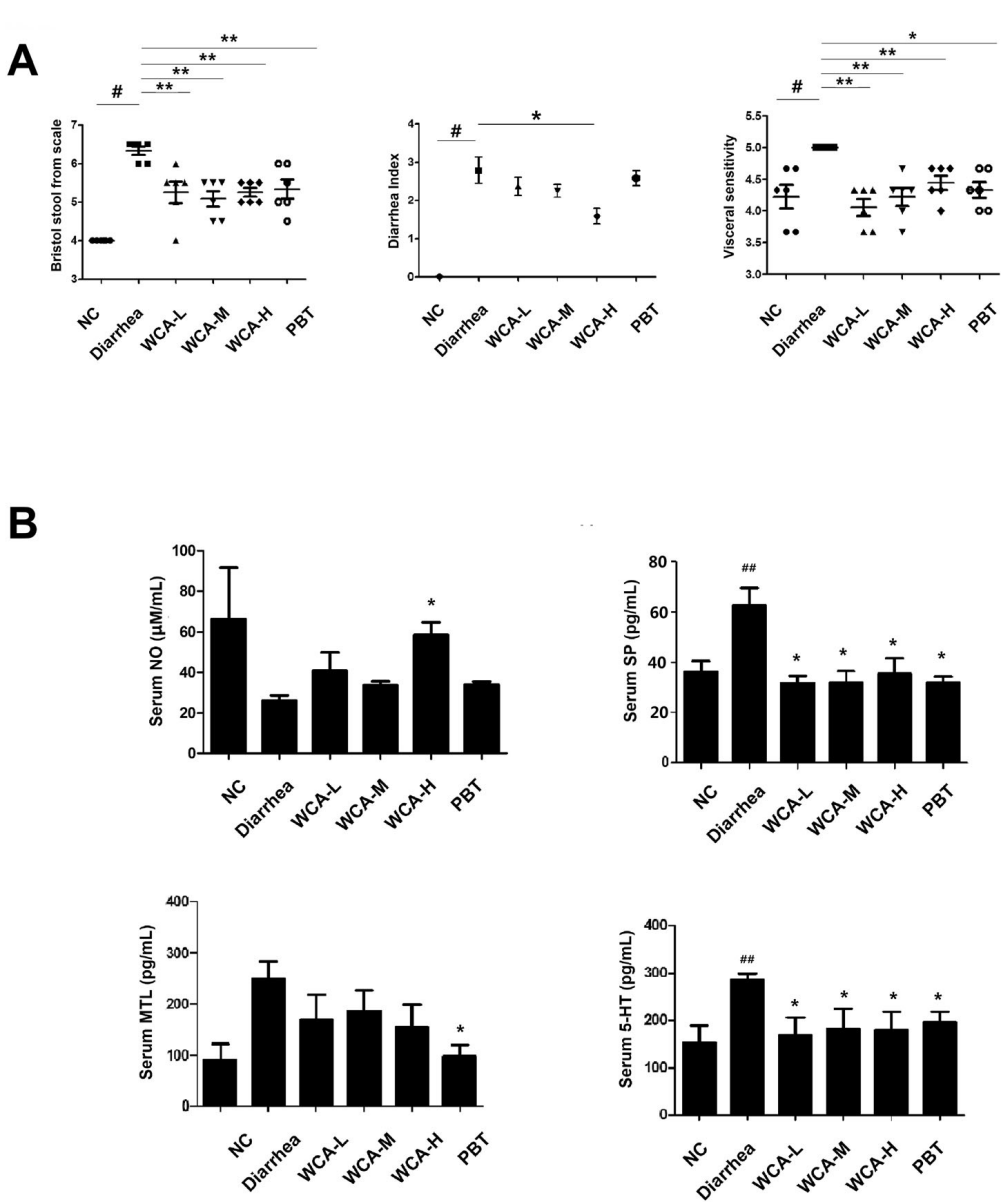
Effects of WCA on faecal property and cytokine expressions in *Folium senna*-induced diarrhoea rats. (A) Bristol stool form scale, diarrhoea index (DI) and Visceral sensitivity scores were calculated. (B) The serum levels of NO, SP, MTL and 5-HT in rats from different experimental groups were analyzed by ELISA. Data are represented as mean ± S.E.M. *n* = 6; ^#^*p* < 0.05 vs. NC; ^##^*p* < 0.01 vs. NC; *p < 0.05 vs. Diarrhoea group; **p < 0.01 vs. diarrhoea group.

**Figure 2.**
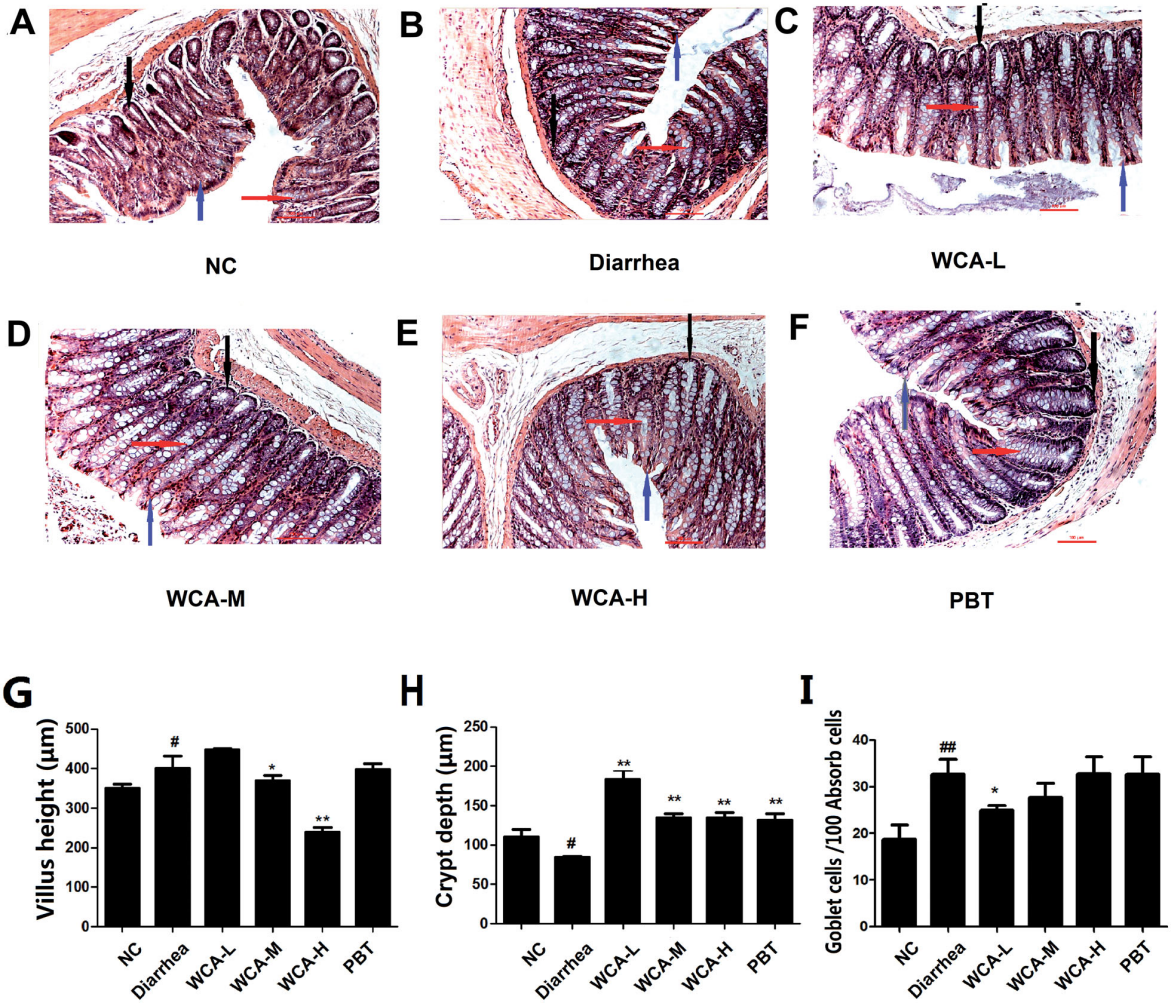
Effect of WCA on pathological changes in *Folium senna*-induced diarrhoea rats.HE staining was performed in colonic tissue from rats. The morphology of colonic structure was captured and labelled for intestinal velvet (blue arrows), Goblet cells (red arrows) and crypt (black arrows). Representative image of diarrhoea group, WCA-L, WCA-M, WCA-H and PBT group were shown. (A–F, *n* = 6). (H) Villus height (µm); (I) Crypt depth (µm); (G) Goblet cells/100 Absorb calls (*n* = 10). Scale bars 100 µm.

To investigate the effect of WCA on diarrhoea, we established the diarrhoea model by given *Folium senna* in rats. As we expected, faeces in diarrhoea group exhibited soft blobs with an unclear cut edge or fluffy pieces with ragged edges and mushy, while in contrast, faeces from NC group appeared smooth and soft like sausages or snakes. Furthermore, after WCA treatment, faecal properties exhibited mass shapes. Consistently, the Bristol stool form scale was high in diarrhoea group (6.3 ± 0.26), but much low in the WCA groups (5.1 ± 0.49) (Figure 1(A), left panel). DI and visceral sensitivity were also highly increased in diarrhoea group (2.79 ± 0.84 and 5.00 ± 0.00) when compared with those in the NC group (0.00 ± 0.00 and 4.22 ± 0.46). However, after WCA administration, DI and visceral sensitivity were both significantly decreased (1.58 ± 0.49 and 4.06 ± 0.33) (Figure 1(A) middle and right panel). As we knew, low levels of NO and high levels of SP, MTL and 5-HT may cause excessive contraction of smooth muscle, leading to diarrhoea (Liu et al. 2019). According to our data, the level of NO was lower (26.16 ± 5.13 μM/mL), and SP (62.65 ± 16.86 pg/mL), MTL (245.64 ± 82.59 pg/mL) and 5-HT (287.93 ± 39.00 pg/mL) were much higher in diarrhoea group than those in NC group (41.37 ± 6.03 μM/mL; 35.84 ± 11.36 pg/mL; 110.11 ± 78.47 pg/mL; 122.78 ± 58.07 pg/mL), all of which were further regulated in WCA groups (58.64 ± 12.37 μM/mL; 31.43 ± 6.82 pg/mL; 193 ± 130.44 pg/mL; 168.34 ± 90.19 pg/mL, Figure 1(B)). As presented in Figure 2(A–F), histological changes were detected in colon tissue by HE staining. Rats in diarrhoea group exhibited significant loss of crypt architecture, degeneration and lengthening of villi (Figure 2(B)), which were much improved in WCA administration (Figure 2(C–E)). The quantified data of colon villus height and crypts depth was showed in Figure 2(G,H). In comparison with those in the NC group, rats in diarrhoea group exhibited increased villus heights (407.06 ± 23.23 μm, *p* < 0.01), as well as less crypts depth in the colon (84.09 ± 0.24 μm, *p* < 0.05). Conversely, treatment with WCA attenuated the colon injury and resulted in a colon condition similar to that observed in the NC group. Specifically, the WCA-M group exhibited shortening villus (240.38 ± 26.69 μm, *p* < 0.01), greater crypts depth (134.27 ± 12.78 μm, *p* < 0.01), compared to those of diarrhoea group. Goblet cells were observed in the lower half of the villi with vacuoles staining and were dispersed among the epithelial cells. The number of goblet cells was significantly higher in diarrhoea group (32.72 ± 7.69, *p* < 0.01) than that of the NC group (18.78 ± 7.92), which was significantly decreased in WCA-L (24.92 ± 2.42, *p* < 0.05) (Figure 2(F)). The effect of PBT on diarrhoea was also analyzed as the positive control, and only the crypts depth was significantly improved when compared with those of diarrhoea group (Figure 2(H)).

### WCA inhibited CDT-induced constipation in rats

**Figure 3.**
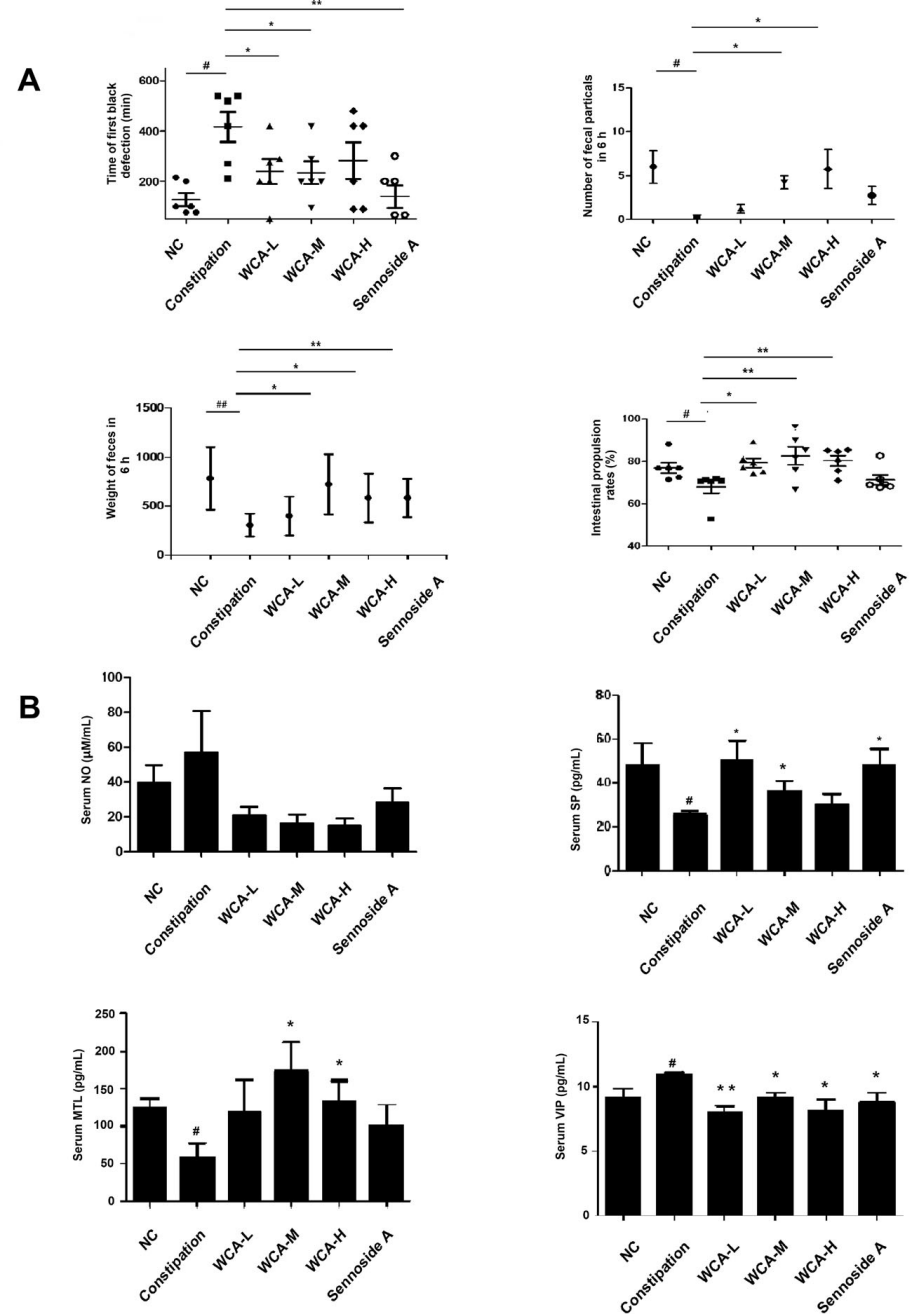
Effects of WCA on faecal properties and cytokine expression in CDT-induced constipation rats. (A) Time of first black defaecation, faecal quantity and weight and intestine propulsion were much recorded and calculated. (B) The serum levels of NO, SP, MTL and VIP in rats were analyzed by ELISA. Data are represented as mean ± S.E.M. *n* = 6; ^#^*p* < 0.05 vs. NC; ^##^*p* < 0.01 vs. NC; *p < 0.05 vs. Constipation group; **p < 0.01 vs. constipation group.

**Figure 4.**
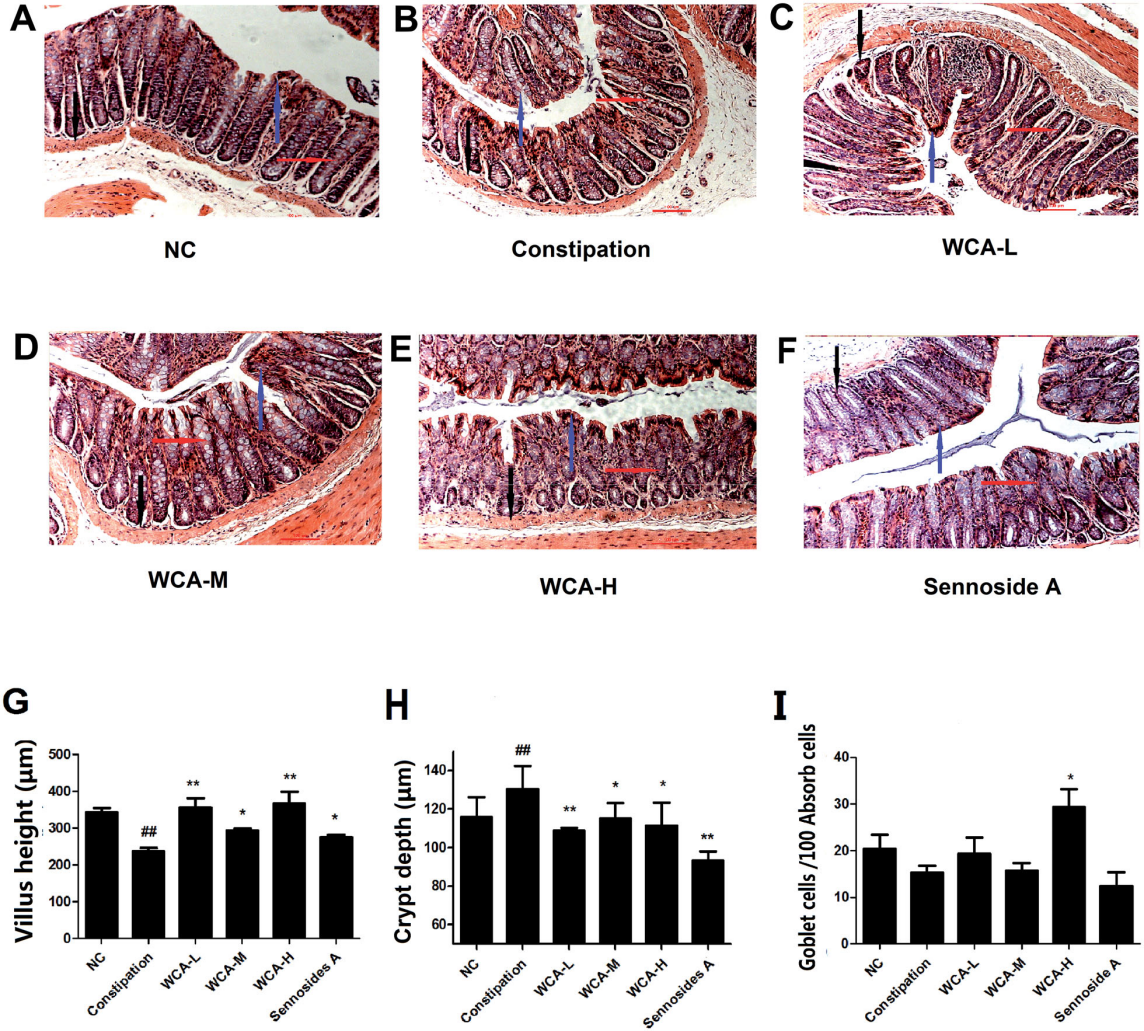
Effect of WCA on pathological changes in CDT-induced constipation rats. HE staining was performed in colonic tissue from rats. The morphology of colonic structure was captured and labelled for intestinal velvet (blue arrows), Goblet cells (red arrows) and crypt (black arrows). Representative image of constipation group, WCA-L, WCA-M, WCA-H and sennoside A group were shown. (A–F, *n* = 6). (H) villus height (µm); (i) crypt depth (µm); (g) goblet cells/100 absorb calls (*n* = 10). Scale bars 100 µm.

We also established a rat constipation model by given CDT, and WCA was then administrated. As shown in Figure 3(A), the time of first black defaecation was highly extended, and the number of faecal particles and their weights in 6 h was obviously decreased in the constipation group, when compared with those in the NC group. And the intestinal propulsive rate was significantly declined in the constipation group as expected when compared with that in the NC group. However, after WCA administration, the time of first black defaecation was significantly decreased from 431.75 ± 32.23 min to 271.56 ± 15.80 min. The number of faecal particles (2.14 ± 0.85 to 5.04 ± 2.14) and their weights (303.96 ± 118.41 mg to 583.82 ± 247.66 mg) was both increased. Furthermore, the intestine propulsive rate was significantly increased from 68.02 ± 7.46 to 82.59 ± 10.28 as well. Levels of NO and VIP were higher (43.42 ± 47.70 μM/mL, 10.8 ± 0.4 pg/mL), and levels of MTL (58.10 ± 46.00 pg/mL) and SP (25.56 ± 3.52 pg/mL) were much lower in constipation group than those in NC group (25.70 ± 19.56 μM/mL; 9.70 ± 1.77 pg/mL; 123.20 ± 37.00 pg/mL; 48.13 ± 21.9 pg/mL). However, the down-regulation of SP (17.54 ± 7.78 μM/mL) and MTL (7.98 ± 1.00 pg/mL) and the up-regulation of NO (172.17 ± 97 pg/mL) and VIP (36.50 ± 9.66 pg/mL) were all inhibited after WCA treatment (Figure 3(B)), indicating the insufficient smooth muscle motility observed in constipation group was much improved by WCA. As presented in Figure 4(A–F), histological differences were detected in colonic tissues by HE staining. The crypts depth was highly increased and villus height was significantly decreased in the constipation group (Figure 4(B)), both of which were much improved in three WCA groups (Figure 4(C–E)). The quantified data are shown in Figure 4(G,H). In comparison with those in the NC group, the villus height in the constipation group was significantly decreased (236.46 ± 24.08 μm, *p* < 0.01), and crypts depth was deepened in the colon (152.28 ± 15.84 μm, *p* < 0.01), indicating an obvious colon injury during constipation. Conversely, treatment with WCA attenuated the colon injury in the colon, which was comparable to those observed in the NC group. Specifically, the WCA-M group exhibited lengthening of villus (366.75 ± 82.99 μm, *p* < 0.05), shallower crypts depth (93.42 ± 29.30 μm, *p* < 0.05), when compared to those of constipation group. The number of goblet cells was lower in the constipation group (15.19 ± 3.52) than that in the NC group (20.33 ± 6.99), which was significantly increased in the WCA-H group (31.49 ± 7.80, *p* < 0.05) (Figure 4(F)). The effect of sennoside A on constipation was also analyzed as the positive control, and only the crypts depth was significantly improved when compared with that of the constipation group (Figure 4(H)).

### Effect of WCA on the basal or ACh-induced strip contraction in rat antrum, jejunum and ileum

**Figure 5.**
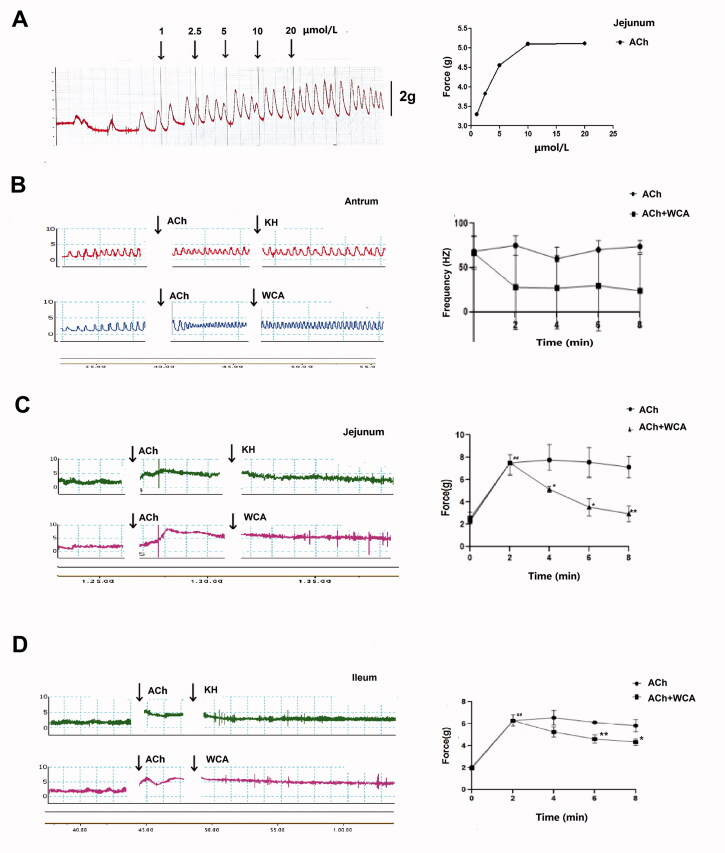
The inhibitory effect of WCA on ACh-induced contractions of rat antrum, jejunum and ileum. (A *n* = 1) Rat jejunum strips were isolated and stimulated with different concentrations of ACh. (B, C, D) Strips from rat antrum (B), jejunum (C) and ileum (D) were isolated and stimulated with ACh (10 µM). After the stable induction of contraction, WCA was added and the contractile force and frequency of spontaneous contractions were recorded. Data are represented as mean ± S.E.M. *n* = 6.

**Figure 6.**
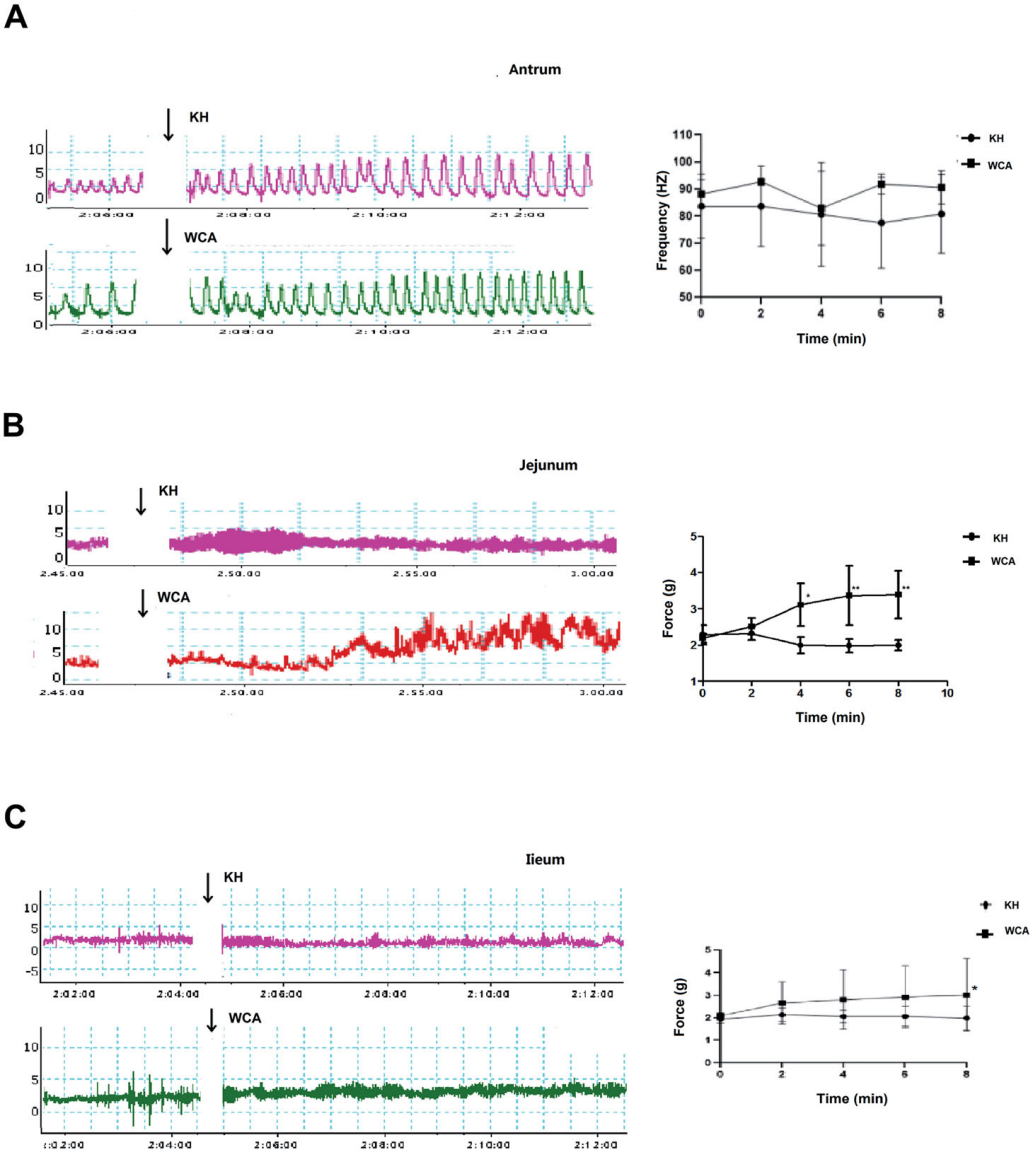
The effects of WCA on contractions of rat antrum, jejunum and ileum. Strips from rat antrum (A), jejunum (B) and ileum (C) were isolated and stimulated with WCA. The contractile force and frequency of spontaneous contractions were recorded. Data are represented as mean ± S.E.M. *n* = 6.

In order to determine the effect of WCA on strip contraction, ACh-induced strip contraction was first established and as shown in Figure 5(A), ACh significantly induced the contraction of jejunum strips in a dose-dependent manner. In the following assays, 10 μmol/L ACh was selected to stimulate the contraction of gastrointestinal muscle strips. The strips of the gastric antrum, intestinal jejunum and ileum were isolated separately. As shown in Figure 5(B), the contraction amplitude of antrum strips was slightly decreased (from the range between 1.23 g and 6.31 g to the range between 1.96 g and 4.92 g), while the frequency was highly increased (from 64.12 ± 9.05 HZ to 69.24 ± 3.78 HZ) when incubation with ACh. However, WCA supplementary suppressed both the effects of contractile amplitude (the range between 1.71 g to 5.17 g) and frequency (23.9 ± 18.76 HZ) in response to ACh. Furthermore, the contractile forces to ACh in the jejunum (7.02 ± 3.94 g) and ileum strips (6.79 ± 2.52 g) were suppressed by WCA supplementary (3.90 ± 1.54 g; 4.41 ± 1.63 g, *p* < 0.05) as well (Figure 5(C,D)). Subsequently, the contractile effect of WCA on gastrointestinal strips alone was assessed. As shown in Figure 6(A), WCA incubation slightly increased the contraction frequency of antrum strips (87.9 ± 1.89 Hz to 92 ± 6.57 Hz, *p* > 0.05), while it has no effect on the contraction amplitude. However, WCA directly increased the contractile force of jejunum and ileum strips (2.02 ± 0.35 g to 3.35 ± 0.23 g; 2.21 ± 0.94 g to 2.97 ± 1.76 g, *p* < 0.05) (Figure 6(B,C)). Taken together, strip assays indicated WCA regulates gastrointestinal contraction in a bidirectional manner, which consistently with our findings on diarrhoea and constipation rats *in vivo.*

### WCA regulated the expressions of ROCK-1 and MLCK in antrum and duodenum

**Figure 7.**
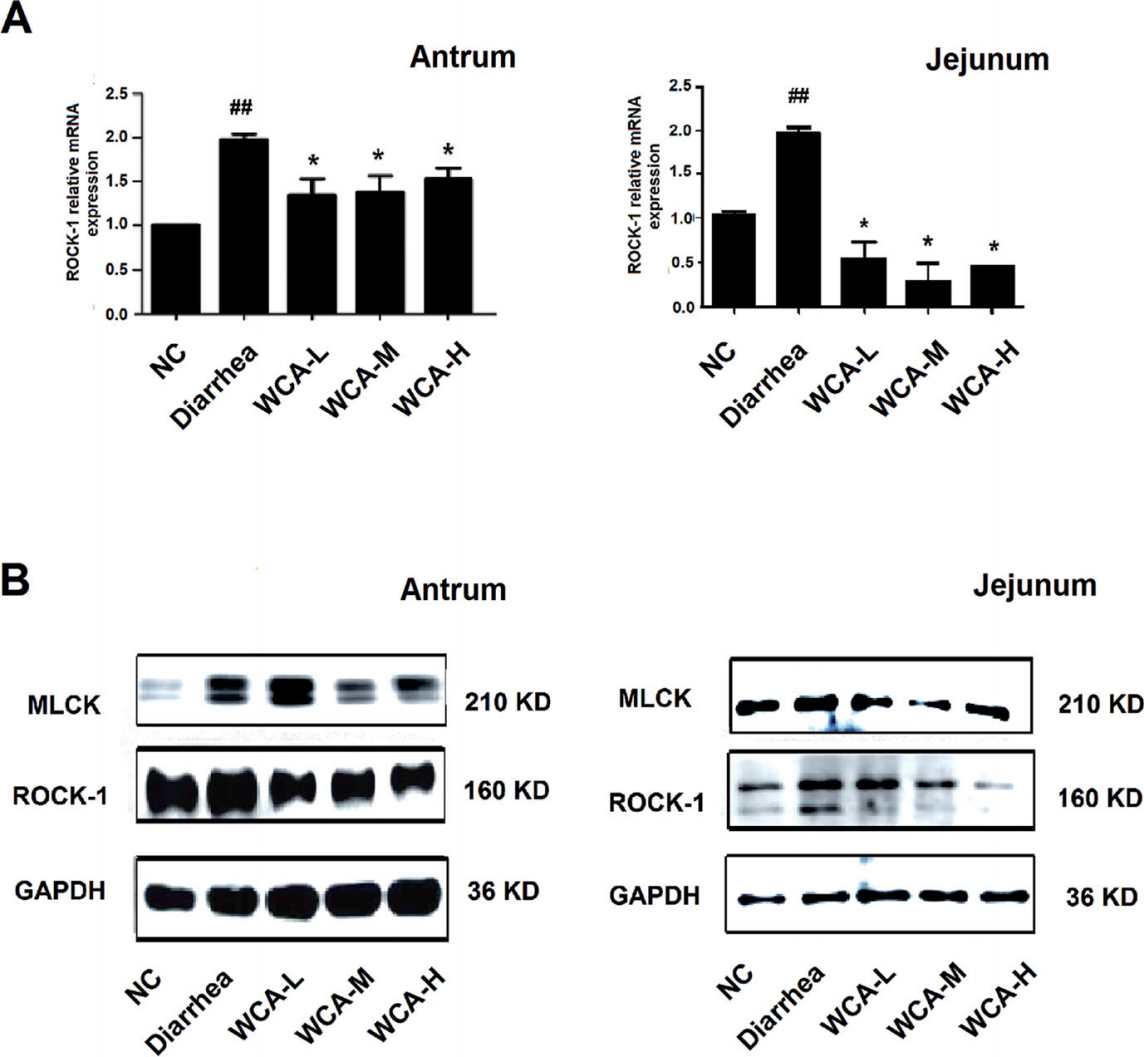
WCA inhibited up-regulation of ROCK-1 and MLCK expressions in diarrhoea rat. (A) Total RNA was extracted and RT-PCR was performed to detect the relative mRNA expression of ROCK-1. (B) Protein was extracted and Western blotting was performed to detect the protein expressions of ROCK-1 and MLCK in antrum and jejunum. The images shown are representative of three independent experiments. Data are represented as mean ± S.E.M. ^##^*p* < 0.01 vs. NC; *p < 0.05 vs. diarrhoea group.

**Figure 8.**
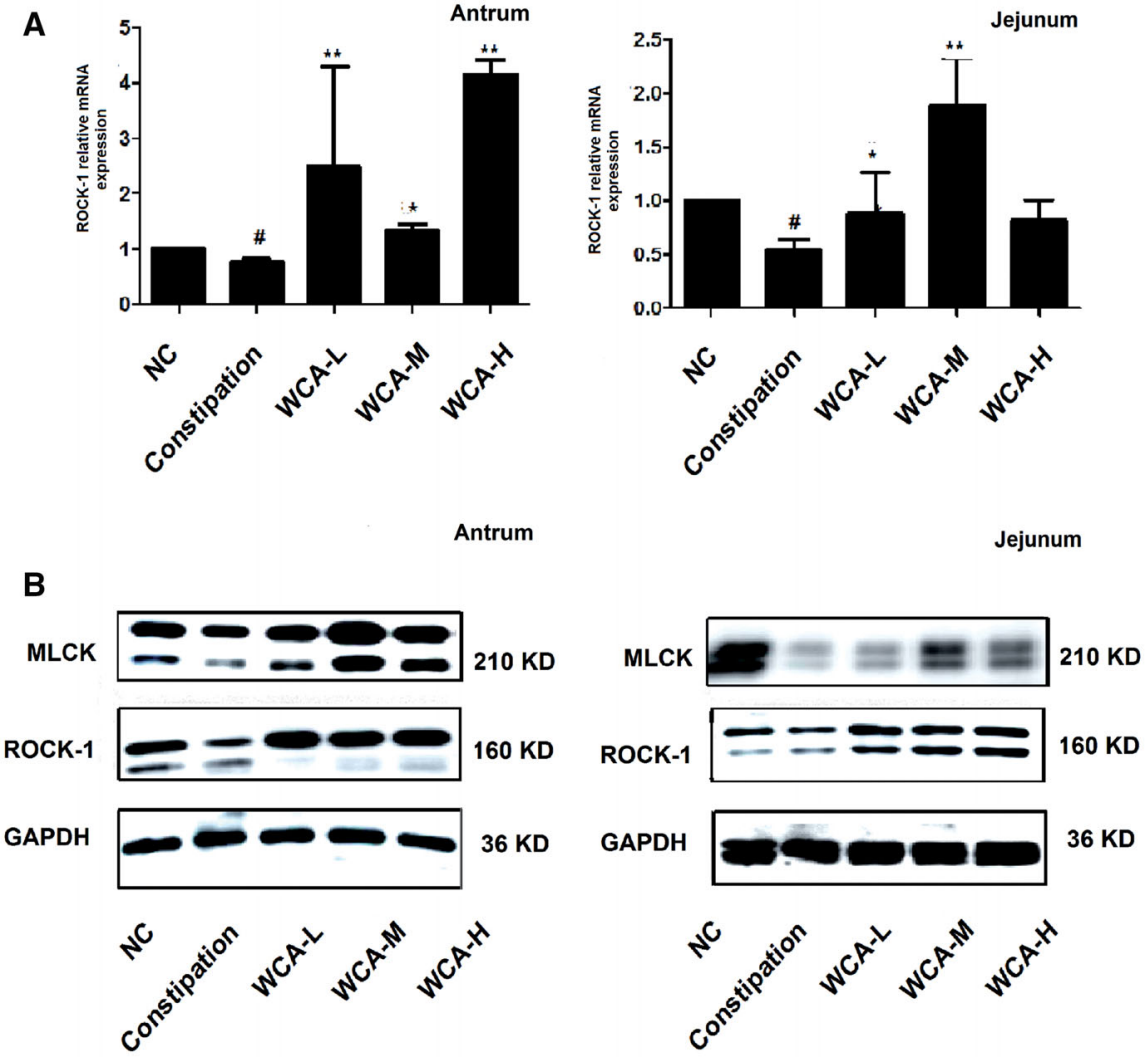
WCA enhanced down-regulation of ROCK-1 and MLCK expression in constipation rats. (A) Total RNA was extracted and RT-PCR was performed to detect the relative mRNA expression of ROCK-1. (B) Protein was extracted and Western blotting was performed to detect the protein expression of ROCK-1 and MLCK in antrum and jejunum. The images shown are representative of three independent experiments. Data are represented as mean ± S.E.M. ^#^*p* < 0.05 vs. NC; *p < 0.05 vs. constipation group; **p < 0.01 vs. constipation group.

ROCK-1 and MLCK are two of the key enzymes that contribute to smooth muscle contraction. High levels of ROCK-1 and MLCK may cause excessive contraction of smooth muscle (Fukata et al. 2001; Patel and Rattan 2007; Kim et al. 2008; Wu et al. 2018). When compared with the expression levels in the NC group, ROCK-1 mRNA and protein expressions and MLCK protein expression were much increased in both antrum and jejunum in diarrhoea rats (Figure 7) but much decreased in Constipation rats (Figure 8), which were consistent with the abnormal regulatory responses to diarrhoea and constipation symptoms. However, the up-regulation of ROCK-1 and MLCK expression in diarrhoea rats and down-regulation in constipation rats were both suppressed by WCA administration, suggesting a bidirectionally effect on the regulation of gastrointestinal motility.

## Discussion

Diarrhoea and constipation are two common gastrointestinal types of IBS, which have opposite symptoms, but both are related to the abnormal gastrointestinal contraction in response to mental and psychological factors (Ohman and Simren 2007). The classical medicine for IBS treatment aims to improve the typical symptoms and thus adopts distinct therapies for diarrhoea and constipation because of the diverse symptoms. For example, 5-HT antagonists (e.g., cisapride and tegaserod) are commonly recommended for persons with diarrhoea (Munjal et al. 2019), while 5-HT agonists (e.g., alosetron) are approved for constipated persons (Niewinna et al. 2019). However, an ideal medication for IBS treatment should be applied with the clinical signs of either diarrhoea or constipation or mixed symptoms, and no single drug offers a comprehensive effect of what is seen in humans with IBS subtypes. It is necessary and important to focus on the regulatory balance between contraction and relaxation in gastrointestinal smooth muscles. On the other hand, WCA has been successfully applied for the prevention and treatment of IBS, although the mechanism is still not clear. Our study found that WCA significantly inhibited both the symptoms of diarrhoea and constipation in rats, which may give evidence of WCA used for IBS patients, not limited to a single symptom.

Traditional Chinese medicine (TCM) has been well-accepted and used for centuries in China. WCA is a herbal TCM prescription that as a sale product has been widely used for patients with gastrointestinal disorders such as indigestion, enteritis and bacterial diseases in the clinic (Liu et al. 2016; Shi et al. 2018). In recent years, experimental observations have indicated that WCA treatment benefitted for diarrhoea symptoms. It has been reported that WCA decreased the number of faeces in castor oil-induced diarrhoea mice (Hu et al. 2009), accelerated the intestinal transit and inhibited the gastric emptying in neostigmine-induced (Qu et al. 2014) or 5-fluorouracil-induced (Chen et al. 2016) diarrhoea mice. Here, in our study, we found that WCA inhibited the increased levels of Bristol stool form scale, DI and visceral sensitivity (Figure 1(A)), as well as down-regulation of serum SP, MTL and 5-HT (Figure 1(B)) in *Folium senna*-induced diarrhoea rats. HE staining also well indicated that WCA obviously repaired the pathological alterations (Figure 2). Consistently, Hu & Tang showed that WCA increased the activity of intestinal digestive enzymes and regulated gastrointestinal hormones in diarrhoea rats as well (Hu and Tang 2012). No experimental data have been reported that WCA improves the symptoms of constipation before. Here, we first showed that WCA inhibited CDT-induced rat constipation, including reduction of time of first black defaecation, an increase of the number and weight of faecal particles, promotion of the intestinal propulsive rate (Figure 3(A)), improvement of the abnormal levels of NO, VIP, SP and MTL (Figure 3(B)) and the pathological alteration (Figure 4). Moreover, we showed that WCA has opposite regulatory effects on the gastrointestinal tract for diarrhoea and constipation, providing evidence of WCA for IBS treatment as a bidirectional drug on both smooth muscle over- and insufficient-contraction along gastrointestinal tract.

Diarrhoea or constipation is closely related to the disorder of gastrointestinal muscle contraction (Sjölund and Ekman 1987; Li et al. 2000). It has been found that the symptoms of the jejunum and ileum hyper-contractions are manifested as diarrhoea, while in contrast the symptoms of the contractile weakness are manifested as constipation (Gershon 2004; Seidl et al. 2009). In our study, we showed that WCA significantly inhibited ACh-induced intestinal contraction in jejunum and ileum (Figure 5(C,D)). Moreover, it also directly promoted the strip contraction in the absence of ACh (Figure 6(B,C)), Intestinal motility is regulated by neurons (Roganovi et al. 2010; Blennerhassett and Lourenssen 2021; Sahakian et al. 2021) and hormones (Ouyang and Cohen 1981). In our study, ACh stimulation in strip assays was used to mimic the induction by neurotransmitters. And the neurotransmitter levels of NO (inhibitory), VIP (inhibitory), SP (excitatory) and 5-HT (excitatory) in rat serum were evaluated. Results showed that SP and 5-HT levels were enhanced and NO level was reduced in Diarrhoea rats (Figure 1(B)). while in contrast SP and 5-HT levels were reduced and NO and VIP levels were enhanced in Constipation rats (Figure 3(B)), when compared with those in the NC group. Both of the abnormal levels of neurotransmitters were significantly blocked after WCA administration (Figures 1(B) and 3(B)), indicating that it may have dual effects of WCA on the balance of contractile behaviours in gastrointestinal smooth muscle.

It has been reported that activation of ROCK-1 and/or MLCK promotes smooth muscle contraction (Fukata et al. 2001; Patel and Rattan 2007; Kim et al. 2008; Wu et al. 2018). Our study showed that the increased ROCK-1 and MLCK expression was easily observed in the antrum and intestine of diarrhoea rats, both of which were significantly inhibited by WCA (Figure 7(B)). In contrast, ROCK-1 and MLCK expressions were decreased in Constipated rats, and further elevated by WCA administration (Figure 8(B)). However, more research is needed to uncover the molecular target and mechanism of WCA in the bidirectional regulation of smooth muscle contraction.

## Conclusions

Taken together, our data showed that WCA alleviates disease symptoms of both rat diarrhoea and constipation in a bidirectional manner, giving evidence of WCA as an attractive drug in the treatment of IBS.
